# Reducing the background of ultra-low-temperature X-ray diffraction data through new methods and advanced materials

**DOI:** 10.1107/S1600576719003078

**Published:** 2019-04-01

**Authors:** Charles James McMonagle, Michael Richard Probert

**Affiliations:** a Chemistry, School of Natural and Environmental Sciences, Newcastle University, Kings Road, Newcastle upon Tyne NE1 7RU, UK

**Keywords:** X-ray diffraction, low-temperature diffraction, cryostats, data collection, technique development

## Abstract

New methods and advanced materials that significantly reduce the background when collecting single-crystal X-ray diffraction data at ultra-low temperatures using a closed-cycle helium refrigerator are presented here. These include a magnetically controlled internal beamstop and a separate internal collimator that together completely remove the scattering contribution to the background from the beryllium vacuum chamber. Additionally, a new radiation shield made from flexible graphite significantly reduces the background and maintains excellent thermal properties. In combination these improvements have led to a sixfold reduction in the average intensity and a 15-fold reduction in peak intensity of the background observed for diffraction experiments conducted with a closed-cycle helium refrigerator. Moreover, access to ultra-low base temperatures, 2.05 K, has been maintained. The design and implementation of these methods are discussed along with a case study of vitamin C to demonstrate the effectiveness of the improvements.

## Abstract 2

New methods to reduce the background when using a closed-cycle helium refrigerator for collecting single-crystal X-ray diffraction data at ultra-low temperatures are presented.

## 1. Introduction   

The XIPHOS diffraction facility, now housed at Newcastle University, was designed to broaden the range of extreme sample environments available in the home laboratory (Probert *et al.*, 2010 ▸). One of the environments targeted in recent years has been the ultra-low-temperature region, <10 K. Open flow coolers (OFCs) using nitrogen gas have made single-crystal X-ray diffraction experiments above 80 K routine (Cosier & Glazer, 1986 ▸). Below this temperature, OFCs using helium gas can typically be used above 20 K; however, they can quickly become prohibitively expensive to run in the laboratory environment (Goeta *et al.*, 1999 ▸; Hardie *et al.*, 1998 ▸). To reliably access lower temperatures the sample must be cooled through a different method. Helium bath and continuous flow cryostats both require liquid helium and are physically not suited to the multi-axis rotation of single-crystal diffraction experiments but can still be used with modifications (Fertey *et al.*, 2007 ▸). Closed-cycle refrigerators (CCRs) offer an alternative. These devices are economical to run and can be mounted onto larger goniometers for single-crystal diffraction experiments. Although more commonplace at central facilities such as Diamond Light Source, ISIS, ESRF and ILL, these systems can still be found in the home laboratory.

XIPHOS I, one of the two XIPHOS instruments, is constructed from a large four-circle Huber Eulerian goniometer with offset chi cradle fitted with a Bruker APEXII CCD area detector. This large goniometer supports a three-stage CCR for accessing ultra-low temperatures (base temperature = 1.86 K). The generator is a direct-drive Mo rotating-anode generator, λ = 0.71073 Å, operating at 5.4 kW, coupled with Helios focusing optics, giving a 120 µm beam at the focal point.

Here we present recently developed methods that significantly reduce the background noise inherent in data collected with the CCR while maintaining access to the base temperature. This has been achieved through a combination of new material selection for the thermal shielding, novel collimation of the primary beam and a beamstop located inside the vacuum chamber. These improvements have allowed weakly diffracting samples, which have previously presented challenges in measuring good counting statistics, to be collected with a clear improvement in the quality of the data obtained. In turn these developments open up new possibilities in samples that can be investigated and a wider range of science that can be explored.

The techniques and materials employed are extendable to a range of sample environments and offer significant advantages in terms of manufacturing and cost, as well as data quality.

## 2. The setup   

The CCR fitted to XIPHOS I is a modified APD 202E Displex cryogenic refrigerator (base temperature = 9 K). This Displex has been enhanced with an additional third Joule–Thompson stage, which allows temperatures down to 1.86 K to be achieved [^4^He ILL Joule–Thomson Cryostat (Bourgeat-Lami *et al.*, 2006 ▸), modifications to APD DE202 head completed by AS Scientific Products Ltd]. In order to reach these temperatures, the sample must be isolated from atmospheric conditions, held under vacuum and additionally thermally isolated from black-body radiation.

**Figure 1:**
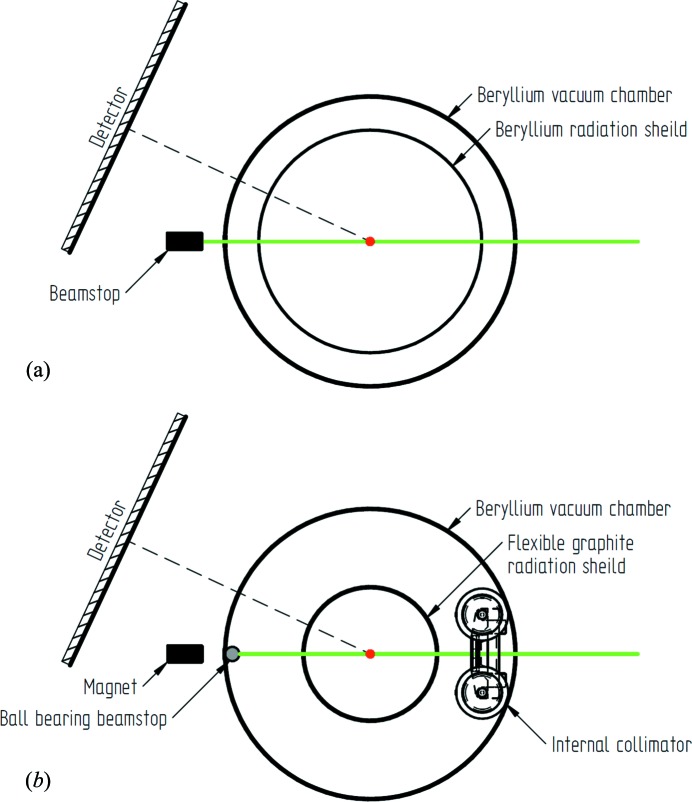
A scale schematic of the setup viewed perpendicular to the primary beam and parallel to the axis of the Displex, with a sample-to-detector distance of 60 mm and 2θ = 25°. The sample is marked by the central red dot and the primary beam in green. (*a*) The original setup: each intersection of the beam with the beryllium of the vacuum chamber and radiation shield produces an intense powder pattern seen on the detector. (*b*) The new setup: the powder pattern from entering the vacuum chamber is removed by the internal collimator and there is no exit pattern because of the ball bearing beamstop. The powder pattern from the flexible graphite is very uniform and more than an order of magnitude less intense than that from the beryllium.

The vacuum chamber is constructed from a cylindrical beryllium shroud. Beryllium, while mostly transparent to X-rays, contaminates the diffraction pattern with a characteristic set of intense powder diffraction rings originating from each intersection with the primary beam [Fig. 1 ▸(*a*)]. Additionally, between the vacuum chamber and the sample it is necessary to have an actively cooled black-body thermal radiation shield, commonly made from beryllium or aluminium foil. This radiation shield contributes a second set of powder rings which results in a complex and highly varying background of overlapping powder diffraction patterns from the four surfaces.

A further problem results from the fact that these diffraction rings are not smooth or consistent from one orientation to the next as the beryllium contains randomly distributed larger crystallites [see Fig. 4(*a*) below]. In turn, this makes simple background subtractions, based on smoothly varying statistics, during data processing inadequate. In the case of weakly diffracting crystals, the irregularities in the background can be of the same order of magnitude as the sample reflection intensities, making the extraction of accurate observed reflection intensities difficult. Methods have previously been developed to improve this situation by collecting data at multiple detector distances in combination with a complex set of ‘masks’ during data reduction to ensure that the intensities from those reflections most affected by scattering from the shrouds are discarded (as implemented in the program *Masquerade*; Coome *et al.*, 2012 ▸).

However, a far more preferable solution would be the reduction or elimination of the parasitic diffraction from the experimental data. Various arrangements of internal beamstop and collimators have previously been developed for cryogenic use (Darovsky *et al.*, 1994 ▸) and sample environments such as pressure cells (Wilkinson *et al.*, 2011 ▸). Replacement of the beryllium for a Kapton-film vacuum chamber is an another approach to controlling the background (Meserschmidt *et al.*, 2003 ▸). The developments presented here offer new, simple and reliable methods to access the ultra-low-temperature region with minimal background.

### 2.1. Beamstop   

**Figure 2:**
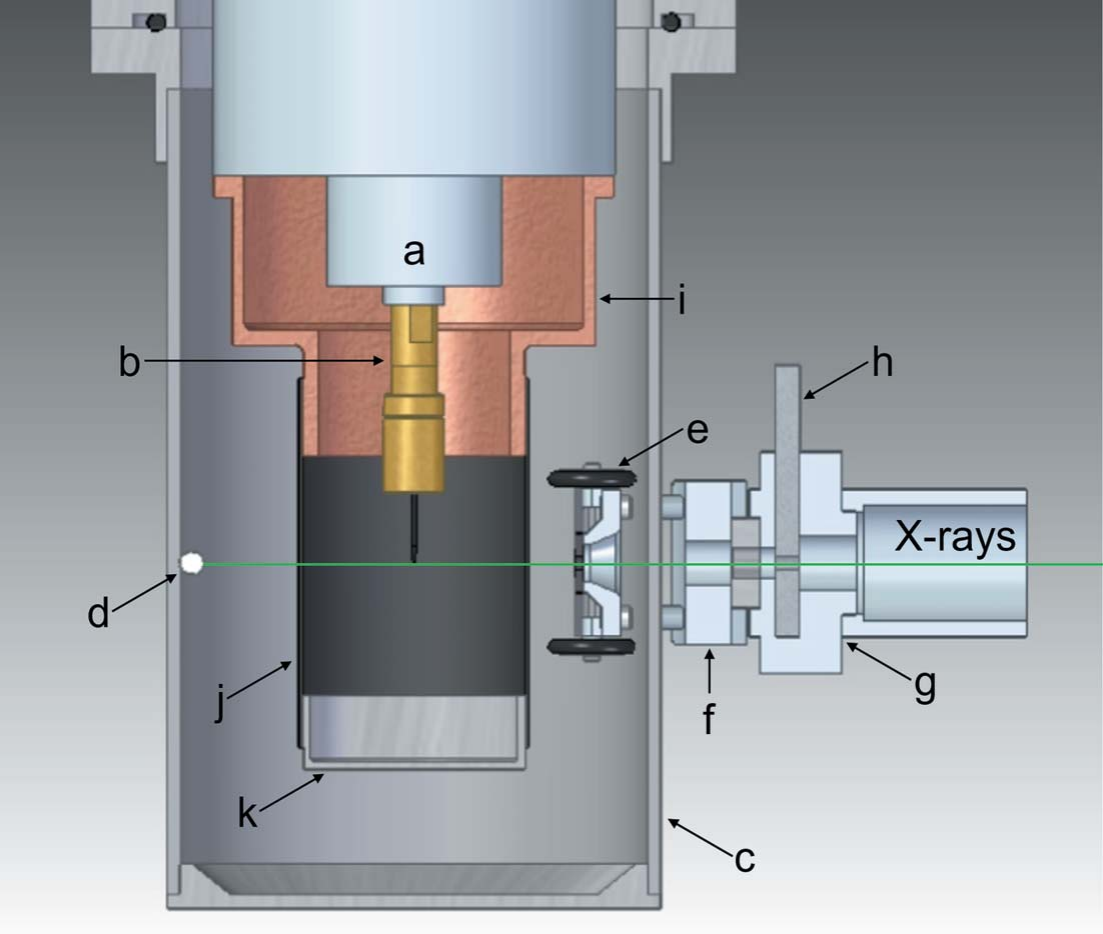
A section view of the new setup as viewed perpendicular to the Displex and primary beam, shown in green. Annotated are (a) the third stage of the modified APD 202E Displex; (b) the brass sample holder with crystal mounted onto a sharpened graphite rod; (c) the beryllium vacuum chamber; (d) the internal ball bearing beamstop; (e) the 3D-printed cart holding the pinhole of the internal collimator; (f) the magnetic clamp; (g) the fixed clamp; (h) the mild steel locking bar; (i) the copper adaptor for the radiation shield; (j) the flexible graphite shroud; (k) the aluminium end cap.

**Figure 3:**
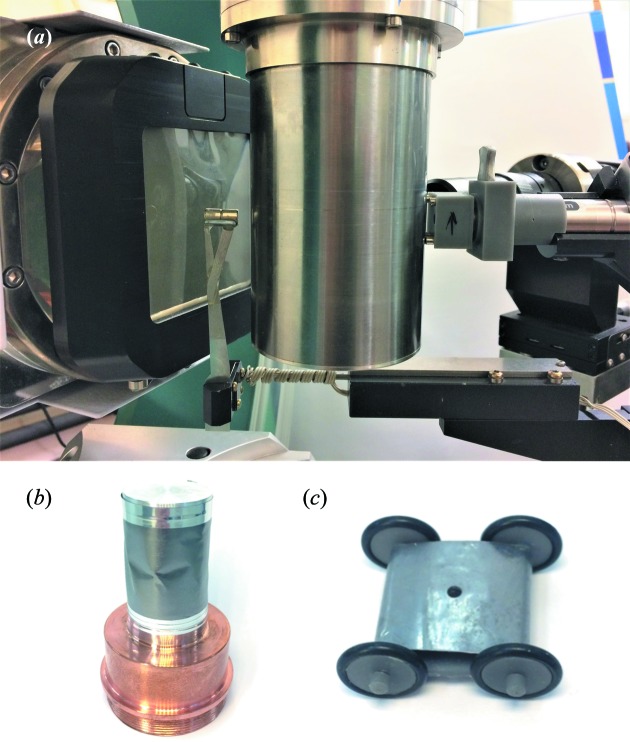
(*a*) Photograph of the new setup on XIPHOS I. Note the magnet in place of the original beamstop to hold the internal beamstop, and the magnetic clamp controlling the internal collimator. (*b*) The new radiation shield with Cu adaptor, flexible graphite shroud and aluminium end cap. (*c*) The 3D-printed cart of the internal collimator. (Images not to scale.)

The most intense contribution to the background was found to be produced from where the primary beam exits the beryllium vacuum chamber, as this is the closest surface to the detector and the scatter from the other surfaces is attenuated by the sample environment itself. The resultant scatter from this surface can be removed by placing the beamstop within the vacuum chamber. This has been achieved with a 1/8′′ carbon steel ball bearing held in place on the inside wall of the vacuum chamber by a high-strength neodymium magnet located at the original beamstop position (Fig. 2 ▸, d). The advantage of this system over other internal beamstop arrangements is its simplicity and self-aligning nature. Here there is no additional restriction on the orientation of the sample: as the Displex is rotated in omega (ω), phi (φ) or chi (χ), the ball bearing simply rolls on the inside of the vacuum chamber to stay aligned with the incident beam. The external magnet utilizes the original beamstop holder with integrated safety circuit and is kept at a distance of ∼5 mm from the vacuum chamber to accommodate any precession of the Displex around the aligned crystal position (Fig. 3 ▸). The external magnet can be detached and used to pick up the ball bearing from the bottom of the vacuum chamber once the system is sealed.

### 2.2. Collimator   

To remove the remaining powder diffraction rings associated with the vacuum chamber as the X-rays enter the Displex, an internal collimator was developed. The internal collimator consists of a 3D-printed cart that holds a 3 mm-diameter lead pinhole, 8 mm from the inside of the vacuum chamber wall (Fig. 2 ▸, e). This provides the collimation required to block all of the contaminating diffraction. The cart has four rubber-banded wheels that allow the collimator to move smoothly on the inside of the vacuum chamber during operation. It is aligned to the beam and held to the vertical side of the vacuum chamber using an external magnetic clamp fixed to the main incident collimator of the diffractometer (Fig. 2 ▸, f–h). The holding power is generated by high-strength neodymium magnets fitted to both the cart and the external clamp.

The clamp is split into two sections, the magnetic and the fixed. The magnetic section of the clamp holds four rod magnets that align and hold the internal collimator in place. The rod magnets are able to slide in the axis of the beam to allow for precession of the Displex. This part also incorporates a larger ring magnet that forms a rotatable coupling with the fixed part of the clamp to allow for rotations in χ. The fixed part of the clamp attaches to the main collimator and aligns the magnetic part to the beam through the rotatable coupling. The two parts of the clamp are held securely together on the insertion of a mild steel bar into the top of the fixed part (Fig. 2 ▸, h). Being able to split the clamp allows the magnetic part of the clamp (Fig. 2 ▸, f) to stay magnetically attached to the internal 3D-printed cart (Fig. 2 ▸, e) to enable both parts of the assembly to remain attached to the vertical wall of the vacuum chamber during sample changes. Additionally the arrangement allows the internal collimator to be easily moved into the operating position once the vacuum chamber is sealed. Further descriptions and details are provided in the supporting information (Section S3), along with an exploded diagram of the collimator assembly (Fig. S2) and CAD drawings (Fig. S3).

With the Displex fitted, movements in χ are limited to 180 ± 22° owing to the vacuum lines and He charge lines linking the Displex to the compressor. Typically, data collection strategies are based on a series of 340° φ scans, as this places the least strain on the goniometer circles. To ensure the alignment of the internal collimator with the primary beam, the vertical wall of the vacuum chamber must be perpendicular to the beam. Therefore, if scans are required where χ ≠ 180°, ω must be set to 0°. These additional restrictions to the movement of the goniometer, in practice, only become an issue for the lowest-symmetry systems.

The combination of the internal collimator and internal beamstop makes the beryllium vacuum chamber essentially invisible to the experiment and so provides no contribution to the background in the diffraction pattern.

### 2.3. Radiation shield   

The remaining background inherent to the Displex is caused by the actively cooled radiation shield. This is a chamber that is attached to the second stage of the Displex and cooled to approximately 12 K. At this temperature there is essentially no emission of black-body radiation from the surface, allowing the sample to be cooled by the third stage to much lower temperatures in thermal isolation. Without a cold radiation shield the base temperature increases to >12 K.

The original radiation shield was made with a beryllium shroud, 50 mm in diameter and 0.25 mm thick. This radiation shield was very effective, allowing the sample to reach the ultimate base temperature of 1.86 K of the Displex, but it caused a very intense background. Replacing the beryllium with aluminium foil, 0.012 mm thick, reduces the intensity of the background, but this comes at the cost of a higher base temperature, ∼3.2 K.

**Figure 4:**
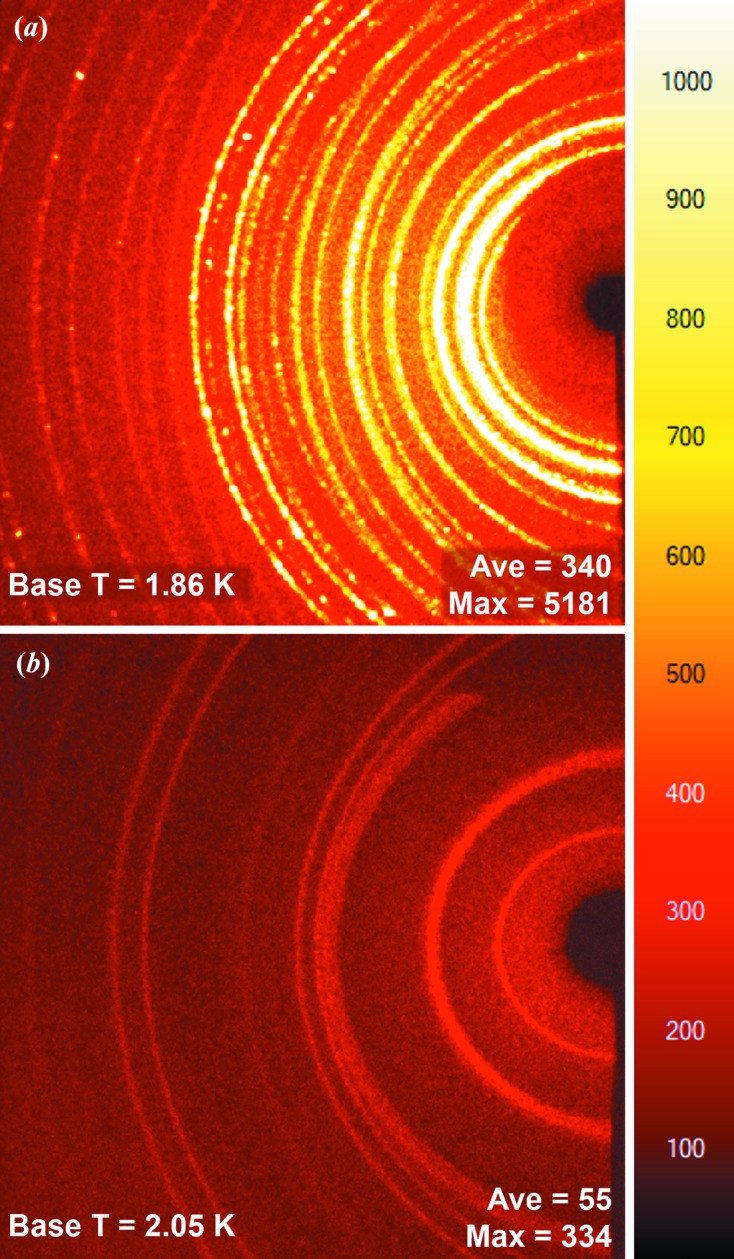
Two frames (20 s exposure time, 0.5° rotation) with the same colour scaling. (*a*) From the original configuration of the beryllium vacuum chamber, beryllium radiation shield and external beamstop. (*b*) From the new setup with the internal collimator, flexible graphite radiation shield and internal beamstop. Insets show the base temperature achievable with the respective setups and the average and maximum intensity on the image.

To address the background and base-temperature issues, a new radiation shield was designed. A copper adaptor reduced the diameter of the shroud to 30 mm to decrease its surface area and mass for optimal cooling (Fig. 2 ▸, i). A large number of materials were then tested for use as the radiation-shield shroud. Ultimately, flexible graphite (0.075 mm, Goodfellow Cambridge Ltd) was found to provide the best combination of thermal conductivity and background scatter. The radiation shield was completed with an aluminium end cap to enclose the sample. The flexible graphite was attached to the copper adaptor and end cap using metallized polyester tape (3M) to give a good thermal union between parts. With this setup the base temperature is 2.05 K, only 0.19 K above the absolute base provided when using a beryllium radiation shield. The flexible graphite does contribute to the background, but the diffracted intensities are an order of magnitude lower than when using the original beryllium radiation shield. In addition, the resulting powder diffraction rings are extremely smooth and uniform, *i.e.* the material contained no large crystallites, allowing simple background subtraction during data reduction [Fig. 4 ▸(*b*) and S4]. This thickness of flexible graphite was calculated to have a transmission rate for Mo *K*α X-rays of 96.5%.

### 2.4. Overview   

The combination of internal collimation, beamstop and reduced scatter thermal shield makes for a drastic reduction in total background. In tests we see a sixfold reduction in the average intensity and a 15-fold reduction in peak intensity in the background (Fig. 4 ▸). Alignment of the internal beamstop and internal collimator is simple, quick and reliable through the extensive use of magnetic control.

These changes have simplified data collection and reduced experiment times because only one detector distance is required as compared to the multiple director distances with the *Masquerade* technique. More significantly, it is now possible to have much longer exposure times without overloading the detector, thereby utilizing the longer collections times to enhance the counting statistics of the reflections. This is particularly important for weakly diffracting samples.

Following the changes to the setup, the low-temperature performance was checked against the phase transition of DyVO_4_ at ∼14 K from tetragonal to orthorhombic on cooling (Göbel & Will, 1972 ▸; Kasten, 1980 ▸). With the new setup the transition was seen to occur within the error of this temperature, verifying the calibration of the apparatus (see Section S1).

## 3. Example of vitamin C   

**Figure 5:**
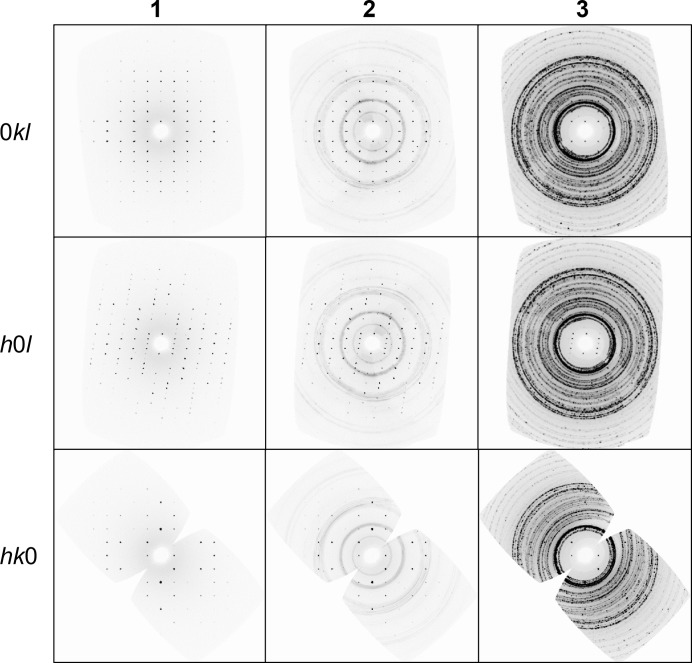
Reconstructed precession images of the (0*kl*), (*h*0*l*) and (*hk*0) planes from collections **1**, **2** and **3**. These clearly show the reduction in the structured background from the improvements described here, **3** versus **2**.

To demonstrate the improvements, three data collections were performed on a crystal of vitamin C (l-ascorbic acid, C_6_H_8_O_6_) (Fig. 5 ▸ and Table 1 ▸). Collection **1** was a control where the sample was mounted onto a MiTeGen loop, centred optically, and data were collected without the vacuum chamber or radiation shield in place. Collection **2** was with all of the changes described above: internal collimator, internal beamstop and flexible graphite radiation shield. And collection **3** was with the original setup: beryllium vacuum chamber, beryllium radiation shield and external beamstop.

All three collections used the same small single crystal (35 × 35 × 10 µm), identical strategies and the same exposure times (20 s per 0.5° frame) to allow direct comparison of intensities and statistics. All data processing was carried out using *APEX3* (Bruker, 2016 ▸) and all structural refinements using *SHELXL* within *Olex^2^* (Sheldrick, 2008*a* ▸, 2015*b* ▸; Dolomanov *et al.*, 2009 ▸). No vacuum was applied in the cases where the vacuum chamber was in place, and all collections were performed at 292 K.

For collection **1** there was no structured background from the sample environment and the unit cell was easily indexed. The data were integrated using the default settings in *SAINT* to a resolution of 0.8 Å and corrected for adsorption in *SADABS* (Bruker, 2014 ▸; Sheldrick, 2008*b* ▸). From the integrated data a structural solution was obtained with *SHELXT* and refined with *SHELXL* (Sheldrick, 2015*a* ▸,*b* ▸). The resulting structure’s final *R*_1_ was 0.0429 [*I* > 2σ(*I*)] with *wR*_2_ of 0.0971 (all data). The stereochemistry of the refined structure was set to match the literature (CSD refcode: LASCAC12; Milanesio *et al.*, 1997 ▸)

Collection **2** simulates the new setup. With the sample environment in place the diffracted intensities are attenuated and as such the resolution was slightly reduced in integration to 0.86 Å. However, the unit cell is still easily indexed and the structure solved with *SHELXT*. The resulting structure refinement is not dissimilar to **1**: final *R*_1_ of 0.0448 [*I* > 2σ(*I*)] and *wR*_2_ of 0.0899 (all data).

Collection **3** presented more problems than **1** or **2**. It was challenging to index the reflections for **3** as the background from the beryllium vacuum chamber and radiation shield is of comparable intensity to the signal from the sample. For integration, a resolution cut-off of 1.3 Å had to be used because of the poor merging statistics and *I*/σ values at higher resolution, again caused by the background. Additionally, it was not possible to solve a structure from the integrated data alone, and only when given a starting model obtained from collection **1** or **2** was it possible to refine a reasonable isotropic structure, 3*.

## 4. Conclusion   

Herein we have presented recently developed methods that have significantly reduced the background when collecting single-crystal X-ray diffraction data at ultra-low temperatures using a closed-cycle helium refrigerator. The magnetically controlled internal beamstop and collimator completely remove any contribution to the background from the beryllium vacuum chamber. Through the use of flexible graphite for the new radiation shield, the remaining background was more than an order of magnitude less intense than that with beryllium while maintaining access to ultra-low base temperatures, 2.05 K. In combination, these improvements have led to a sixfold reduction in the average intensity and a 15-fold reduction in peak intensity for the background.

As an example of these improvements we have tested a very small crystal of vitamin C as a weakly diffracting sample. With the original setup, the unit cell could be indexed but a structural solution could not be obtained. The structure could only be refined against a pre-existing model determined separately. When data were collected using the new setup the structure was easily solved and the refinement resulted in final statistics similar to the control experiment where there was no sample environment in place.

Phase transitions commonly occur on cooling; therefore, it is vital to be able to solve and refine structural data from low-temperature data independently. The presented example shows that this is now possible for a weakly diffracting sample, opening up new possibilities for the type of samples that can be investigated and allowing a wider range of science to be explored.

The techniques and materials employed are extendable to a range of sample environments and offer significant advantages in manufacturing and cost terms, as well as data quality. Some of the techniques are very simple and inexpensive to implement, and all have been very reliable to operate. This setup is now the standard for data collections on XIPHOS I, with very promising results.

## Supplementary Material

Crystal structure: contains datablock(s) Collection_1_vitamin_C, Collection_2_vitamin_C, Collection_3_vitamin_C. DOI: 10.1107/S1600576719003078/kc5091sup1.cif

Structure factors: contains datablock(s) Collection_1_vitamin_C. DOI: 10.1107/S1600576719003078/kc5091Collection_1_vitamin_Csup2.hkl

Structure factors: contains datablock(s) Collection_2_vitamin_C. DOI: 10.1107/S1600576719003078/kc5091Collection_2_vitamin_Csup3.hkl

Structure factors: contains datablock(s) Collection_3_vitamin_C. DOI: 10.1107/S1600576719003078/kc5091Collection_3_vitamin_Csup4.hkl

A video of the setup with a transparent vacuum chamber. DOI: 10.1107/S1600576719003078/kc5091sup5.mpg

Supporting information file. DOI: 10.1107/S1600576719003078/kc5091sup6.pdf

CCDC references: 1900179, 1900180, 1900181

## Figures and Tables

**Table 1:** Crystallographic data for vitamin C collections **1**, **2** and **3** For all structures: C_6_H_8_O_6_, *M*_r_ = 176.12, monoclinic, *P*2_1_, *Z* = 4. All of the experiments were carried out at 292 K with Mo *K*α radiation using a Bruker APEXII CCD and the same crystal, 0.035 × 0.035 × 0.01 mm.

	**1**	**2**	3*
*a* (Å)	6.4213 (2)	6.4196 (2)	6.4259 (12)
*b* (Å)	6.3622 (2)	6.3619 (3)	6.3637 (12)
*c* (Å)	17.1606 (6)	17.1568 (6)	17.168 (4)
β (°)	99.355 (3)	99.356 (3)	99.368 (15)
*V* (Å^3^)	691.75 (4)	691.38 (5)	692.7 (2)
Refections collected	7025	6127	2026
Independent reflections	2609	2183	627
*I* > 2σ(*I*)	1874	1423	444
Completeness (%)	94	95	98
*d*_min_ (Å)	0.80	0.86	1.30
Mean *I*/σ	18.1	12.8	13.6
*R*_int_ (%)	4.5	6.6	7.9
*R*[*F* ^2^ > 2σ(*F* ^2^)], *wR*(*F* ^2^) (%)	4.1, 8.8	4.4, 8.3	6.2, 16.9
No. of parameters	225	225	105
Δρ_max_, Δρ_min_ (e Å^−3^)	0.23, −0.23	0.19, −0.22	0.22, −0.22

*Parameters related to the refined structure for collection **3** were obtained by using the structure refined in **1** as a starting model.
