# Supplementary material for: Reducing the background of ultra-low-temperature X-ray diffraction data through new methods and advanced materials
Source: J Appl Crystallogr. 2019 Apr 1;52(Pt 2):445–50. doi: 10.1107/S1600576719003078 (PMC6448682; doi:10.1107/S1600576719003078)
Supplement: Supplementary file 6 [file j-52-00445-sup6.pdf]

JOURNAL OF APPLIED CRYSTALLOGRAPHY

Volume 52 (2019)

Supporting information for article:

Reducing the background of ultra-low-temperature X-ray diffraction data through new methods and advanced materials

Charles James McMonagle and Michael Richard Probert

S1. Temperature calibration

The temperature of the Displex is controlled with a Lakeshore 340 controller, with measurements inside the head being performed with a Lakeshore DT-470-CO-13 diode. Upon installation this apparatus was checked for calibration with a number of low temperature phase transition materials. With all of the changes that have been implemented over this update the low temperature performance was checked against the phase transition of $DyVO_4$. This was the lowest transition material used in the original testing.

$DyVO_4$ undergoes a tetragonal to orthorhombic phase transition on cooling.at ~14 K. (Göbel & Will, 1972; Kasten, 1980) Here, this was observed directly from the unit cell parameters at the transition. From our measurements the Lakeshore DT-470-CO-13 diode is reading to within 0.5 K of the transition temperature verifying the calibration of the apparatus.

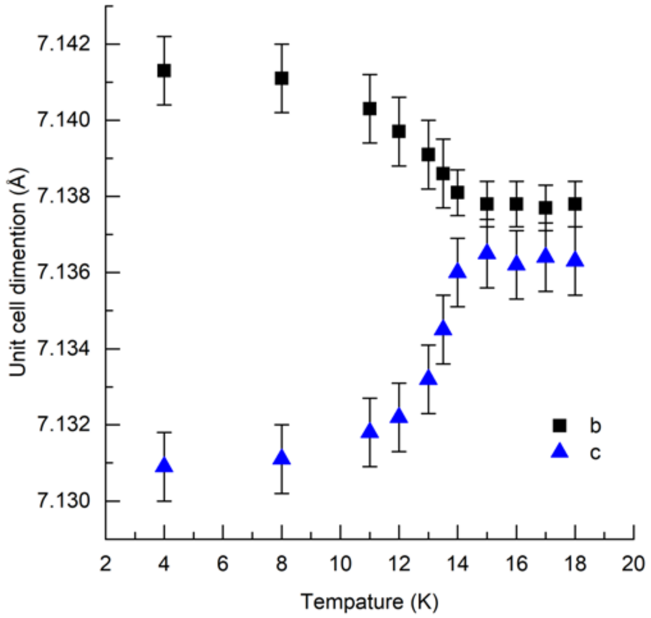

Figure S1 $DyVO_4$ tetragonal to orthorhombic phase transition in line with the literature values of 13.8 K. following unit cell axes the b and c axes are equidistant within a 3σ error until 14 K where upon they split distinctly.

S2. Additive Manufacture

To quickly and accurately produce the parts required for the internal collimator 3D printing was extensively used for prototyping and the final parts. The 3D printer used was a Formlabs Form 2 stereolithography (SLA) 3D printer that uses a laser to cure solid isotropic parts from a liquid photopolymer resin, https://formlabs.com/3d-printers/form-2/. The resin used was the grey standard PMMA resin, FLGPGR04.

S3. Design of internal collimator

S3.1. 3D printed cart

The cart is based on a rectangular design with wheels and magnets at all four corners. The collimation is provided by the 3 mm diameter lead pinhole that is held 8 mm from the inside of the vacuum chamber wall. This thick lead pinhole blocks the most intense low-angle diffraction from vacuum chamber. Higher angle diffraction is blocked by the additional lead shielding covering the cart. This lead is much thinner that the primary pinhole to reduce the weight of the cart but is more than adequate to block the high-angle diffraction from the beryllium. Together this configuration blocks all of the diffraction from the beryllium vacuum chamber while providing a low profile for the internal cart. The 12 mm wheel size was chosen to be the largest that would comfortably fit between the vacuum chamber and radiation shield to reduce the rolling resistance. The wheels are constructed from a 3D printed rim onto which a nitrile rubber O-ring is seated. Stainless steel dress makers pins were used for the axels and a 3D printed hubcap complete the assembly. 3 mm diameter, 4 mm N42 neodymium rod magnets are fitted to each corner for the magnetic control.

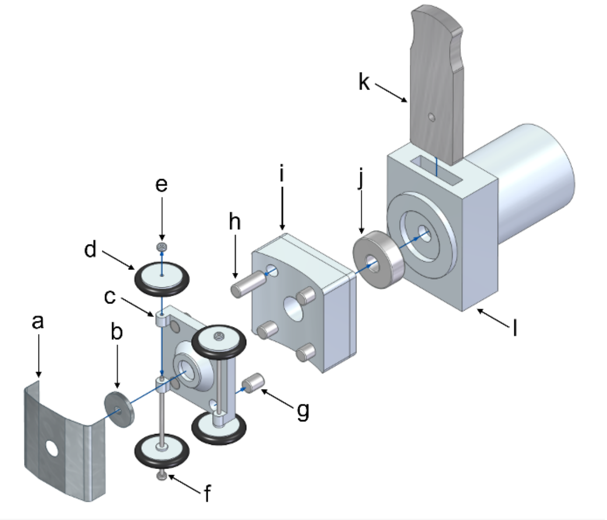

Figure S2 An exploded view of the internal collimator and external clamp assemblies. Annotated are: (a) Lead shielding for cart; (b) lead 3 mm pinhole; (c) 3D printed cart chassis; (d) 3D printed rim with nitrile O-ring tires to give a 12 mm diameter wheel; (e) plastic hubcap; (f) stainless steel axle; (g) 3 mm diameter, 4 mm N42 neodymium magnets; (h) 3 mm diameter, 13 mm N42 neodymium magnets; (i) 3D printed magnetic clamp body; (j) 12 mm OD, 4 mm ID, N42 neodymium ring magnet that is glued to magnetic clamp body; (k) mild steel locking bar; (l) fixed clamp that attaches to the main collimator.

S3.2. The clamp

The clamp is split into two sections, the magnetic and the fixed, to accommodate for rotations in χ and the precession of the Displex. The magnetic part of the clamp has four 3 mm diameter, 13 mm N42 neodymium rod magnets that are able to slide in the axis of the beam to allow for precession of the Displex, and a 12 mm OD, 4 mm ID, N42 neodymium ring magnet is permanently glued to the back of this. The ring magnet forms a rotatable coupling with the fixed part of the clamp that allows for rotations in χ. The fixed part of the clamp attaches to the main collimator and aligns the magnetic part to the beam through the rotatable coupling. The two parts of the clamp are held securely together on the insertion of a mild steel bar into the fixed part as the ring magnet is attracted to it.

The external portion of the collimator is the part that is the least interchangeable between instruments. This is where accurate 3D modelling and rapid prototyping afforded with CAD and 3D printing are invaluable.

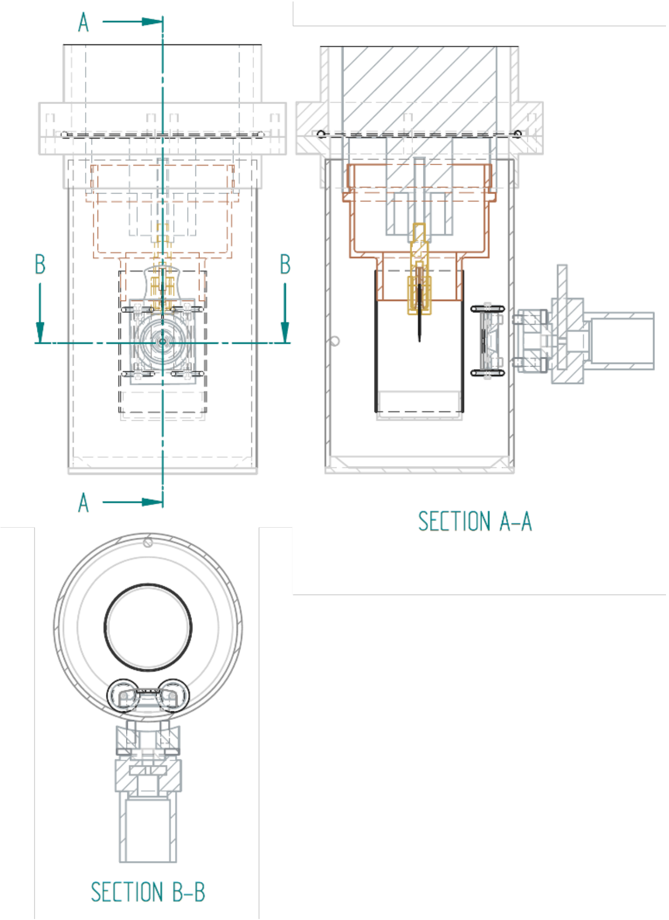

Figure S3 CAD drawing of the head of the Displex and new set-up along with two section views. Section A-A, perpendicular to the Displex and primary beam. Section B-B, parallel to the Displex and perpendicular to the primary beam.

S4. Integrated background

The images of the background shown in Fig 4 integrated in 0.05° steps from 2-50° using APEX3

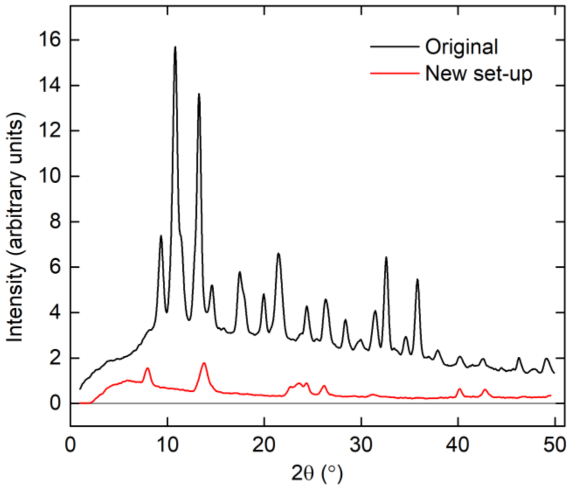

Figure S4 The background of the original and new set-up shown from 2-50° of 2θ, λ = 0.71073 Å.
